# Comparison of Aortic Annulus Diameter Measurement between Multi-Detector Computed Tomography and Echocardiography: A Meta-Analysis

**DOI:** 10.1371/journal.pone.0058729

**Published:** 2013-03-14

**Authors:** Ruifang Zhang, Yi Song, Yuanyuan Zhou, Lulu Sun

**Affiliations:** Department of Ultrasound, the First Affiliated Hospital of Zhengzhou University, Zhengzhou, Henan Province, China; Brigham & Women’s Hospital, and Harvard Medical School, United States of America

## Abstract

**Background and Purpose:**

Accurate measurement of aortic annulus diameter is crucial for choosing suitable prosthetic size for transcatheter aortic valve implantation (TAVI). Several imaging methods are available for the measurement, but significant variability between different modalities has been observed. The purpose of this study was to systematically compare the measurements of aortic annulus diameter between multi-detector computed tomography (MDCT), transthoracic echocardiography (TTE), and transesophegeal echocardiography (TEE).

**Methods:**

PubMed and EMBASE databases between January 2000 and January 2012 were searched. We extracted data from eligible studies evaluating the aortic annulus diameter by MDCT and echocardiography (TTE, TEE, or both). We performed a random-effects meta-analysis to calculate the weighted mean differences of aortic annulus diameter measurement between MDCT, TTE, and TEE.

**Results:**

A total of 10 eligible studies involving 581 subjects with aortic valve stenosis were included. Aortic annulus diameter measured on coronal view by MDCT (25.3±0.52 mm) was respectively larger than that measured on sagittal view by MDCT (22.7±0.37 mm), TTE (22.6±0.28 mm), and TEE (23.1±0.32 mm). The weighted mean difference of aortic annulus diameter between coronal view by MDCT and TTE these two methods was 2.97 mm, followed by the weighted mean difference of 2.53 mm between coronal view and sagittal view by MDCT, and the mean difference of 1.74 mm between coronal view on MDCT and TEE (*P*<0.0001 for all). The weighted mean difference of aortic annulus diameter measurement between TEE and TTE was significant but somewhat small (0.45 mm, *P* = 0.007).

**Conclusion:**

Aortic annulus diameter measured on coronal view by MDCT was robustly and significantly larger than that obtained on sagittal view by MDCT, TTE, or TEE. Such variability of aortic annulus diameter measurement by different imaging modalities cannot be ignored when developing optimal strategies for selection of prosthetic valve size in TAVI.

## Introduction

Transcatheter aortic valve implantation (TAVI) has recently become an alternative to surgical aortic replacement in patients with severe aortic valve stenosis (AS), who are at a high surgical risk or contraindication to conventional aortic valve replacement surgery [1], [2], [3]. For TAVI, accurate measurement of aortic annulus diameter is crucial for choosing suitable prosthetic size. Aortic annulus diameter has been evaluated usually by transthoracic echocardiography (TTE) and transesophegeal echocardiography (TEE). By providing multiple-dimensional information of anatomical shape, multi-detector computed tomography (MDCT) has been also applied for accurate assessment of aortic annulus. Previous studies have assessed its performance by conducting simultaneous assessment of aortic annulus by TTE, TEE and MDCT in AS patients [4], [5] and showed substantial variability in the measurements of aortic annulus diameter between those modalities across studies. For instance, the mean difference of aortic annulus diameter could be as large as 4.1 mm between TTE and MDCT [4] and ranged from 1.4 mm to 4.0 mm between TTE and TEE [6]. Furthermore, some studies showed that aortic annulus diameter measured by MDCT was significantly larger than that measured by TTE or TEE [7], [8] whereas some reported the largest aortic annulus detected by TEE [6]. In clinical practice, a slight difference in the measurement of aortic annulus diameter has a significant impact on valve sizing recommendations and even the procedure decisions [9]. Choosing a larger or smaller size of prosthetic valve could eventually lead to paravalvular leakage, valve embolization, and prosthetic mismatch after procedure [10]. In the absence of gold standard for aortic valve sizing, it is important to learn the sources of variability in the measurement of aortic annulus diameter between different imaging modalities. To provide useful information to help therapeutic decision making, we therefore conducted a systematic review and meta-analysis to compare the overall mean differences in aortic annulus diameter measurement between TTE, TEE and MDCT in AS patients.

## Materials and Methods

### Search Strategy

We searched the PubMed and EMBASE databases between January 2000 and January 2012 with the following keywords: “aortic annulus”, “transthoracic echocardiography”, “transesophegeal echocardiography”, “multi-detector computed tomography”, “transcatheter aortic valve implantation” and “aortic valve stenosis”. The search was restricted to human studies published in English. References of review articles were also searched. Selected studies were individually examined to exclude studies with potentially duplicate and overlapping data. Abstracts without full-text publication, reviews, editorials or letters were excluded.

### Study Selection and Data Extraction

Two reviewers (Zhang R and Sun L) independently searched and retrieved the full text of relevant articles. Discrepancy was resolved by group discussion. In each study, the TTE, TEE, and MDCT measurements of aortic annulus diameter were based on the visualization of the most caudal leaflet hinge points. For MDCT, we chose three widely-performed views -sagittal view, coronal view or three chamber view- on which aortic annulus diameter could be directly measured. Our inclusion criteria of this meta-analysis were listed as follows: (a) studies evaluating the aortic annulus diameter by MDCT and echocardiography (TTE, TEE, or both) were included; (b) the use of CT scanners with a minimum of 16 detector rows; and (c) the aortic annulus diameter measured by MDCT were on sagittal view, coronal view or three chamber view. Articles that did not present relevant data for meta-analysis were not included.

Information on the first author, year of publication, study population, sample size, mean age, background diseases, and methods of aortic annulus measurement was extracted from each included study. The means and standard deviations of aortic annulus diameter by TTE, TEE, and on coronal view, sagittal view or three chamber view by MDCT were obtained from the text, tables, or graphs in each article. For studies with unavailable or incomplete information, the corresponding authors were contacted on 3 occasions over 1 month to complete the missing information. Two of the 6 contacted authors provided the additional data.

In addition, only two studies had a standard surgical gauge measurement of aortic annulus during surgical aortic valve replacement. We also extracted surgical measurement data from those studies in secondary analysis.

### Data Synthesis and Statistical Analysis

Meta-analysis was performed using DerSimonian and Laird’s random-effects model in which each study is weighted by the inverse of the sum of within-study plus between-study variance [11]. The weighted mean differences and 95% confidence intervals of aortic annulus between TTE, TEE, and MDCT were calculated. Results are presented in figures and tables, with the mean and confidence interval for each study being presented, as well as the summary values obtained from the meta-analysis. Between-study heterogeneity was assessed using Cochrane’s *Q* test and the *I* ^2^ test [12]. The percentages of *I^2^* around 25% (*I^2^* = 25), 50% (*I^2^* = 50), and 75% (*I^2^* = 75) indicate low, medium, and high heterogeneity, respectively [12]. We assessed publication bias using visual inspection of Begg’s modified funnel plots, in which the mean difference was plotted against its standard error from each study. Publication bias was also assessed by Begg’s adjusted rank correlation test [13] and Egger’s regression asymmetry test [14]. All analyses were performed using the STATA statistical software (version 10.1, STATA Corp., College Station, Texas). *P*-values of less than 0.05 were considered statistically significant.

## Results

**Figure 1:**
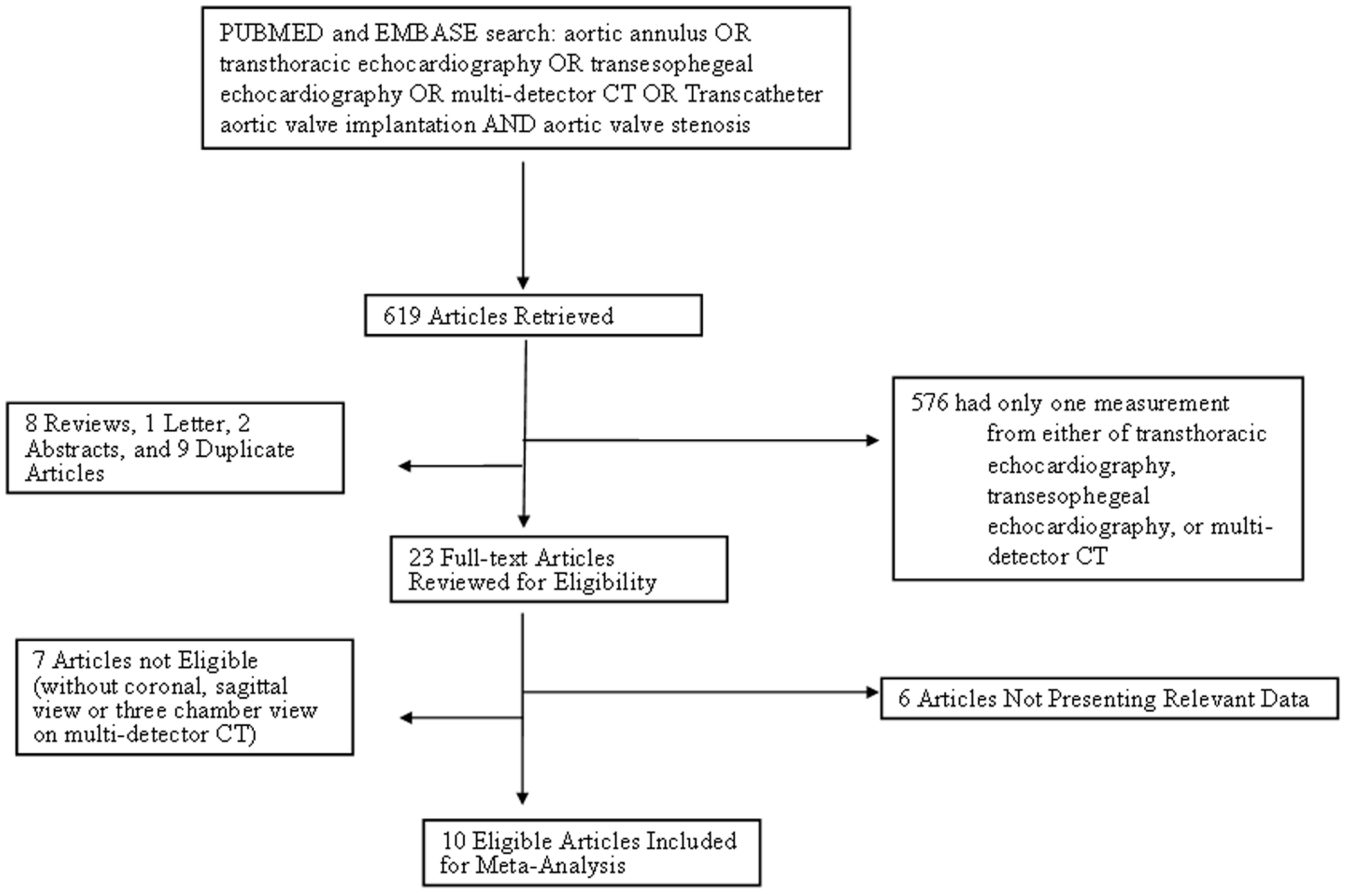
Flowchart of study selection.

In total, 619 titles were screened for relevance, of which 16 articles were considered eligible, including 10 studies with available data (**Figure 1**). Hand-search yielded 3 eligible articles. All individuals in these studies (n = 581) were AS patients. Of these 10 studies [5], [9], [15], [16], [17], [18], [19], [20], [21], [22], 8 studies performed both TTE and MDCT [5], [9], [15], [17], [19], [20], [21], [22]and other 8 studies performed both TEE and MDCT [5], [9], [15], [16], [18], [19], [20], [22]. **Table 1** shows basic characteristics of the 10 included studies.

**Table 1:** Basic characteristics of the 10 included studies involving 581 patients with aortic valve stenosis.

Author	Country	Year of publication	Numberof patients	Meanage (y)	Sex, men (%)	View on TTE	View on TEE	View on MDCT	Measurementphase	Surgical measurement
Wood [22]	Canada	2009	26	82±9	Unavailable (NA)	Para-sternallong axis	Long axis	Coronal andSagittal view	End-diastole	No
Messika-Zeitoun[5]	France	2010	45	80±8	58	Para-sternallong axis	Long axis	Three chamberview	Mid-systole	No
Delgado [17]	Netherlands	2010	53	80±8	55	Para-sternallong axis	NA	Coronal andSagittal view	NA	No
Hutter [9]	Germany	2010	187	81±7	37	Para-sternallong axis	Long axis	Three chamberview	NA	No
Altiok [15]	Germany	2011	49	81±7	33	Para-sternallong axis	Long axis	Coronal andSagittal view	End-diastole	No
Dashkevich [16]	Germany	2011	33	77±8	55	NA	Long axis	Coronal andSagittal view	End-diastole	Yes
Koos [18]	Germany	2011	58	83±6	26	NA	Long axis	Coronal andSagittal view	Diastole	No
Mizia-Stec [20]	Poland	2011	20	69±6	85	Para-sternallong axis	Long axis	Coronal andSagittal view	NA	Yes
Mesa Rubio [19]	Spain	2011	40	77±4	54	Para-sternallong axis	Long axis	Coronal andSagittal view	End-diastole	No
Tzikas [21]	Netherlands	2011	70	82	51	Para-sternallong axis	NA	Coronal andSagittal view	Mid-systole	No

AS: Aortic valve stenosis; NA: Unavailable; TTE: transthoracic echocardiography; TEE: transesophegeal echocardiography; MDCT: multi-detector computed tomography.

**Figure 2:**
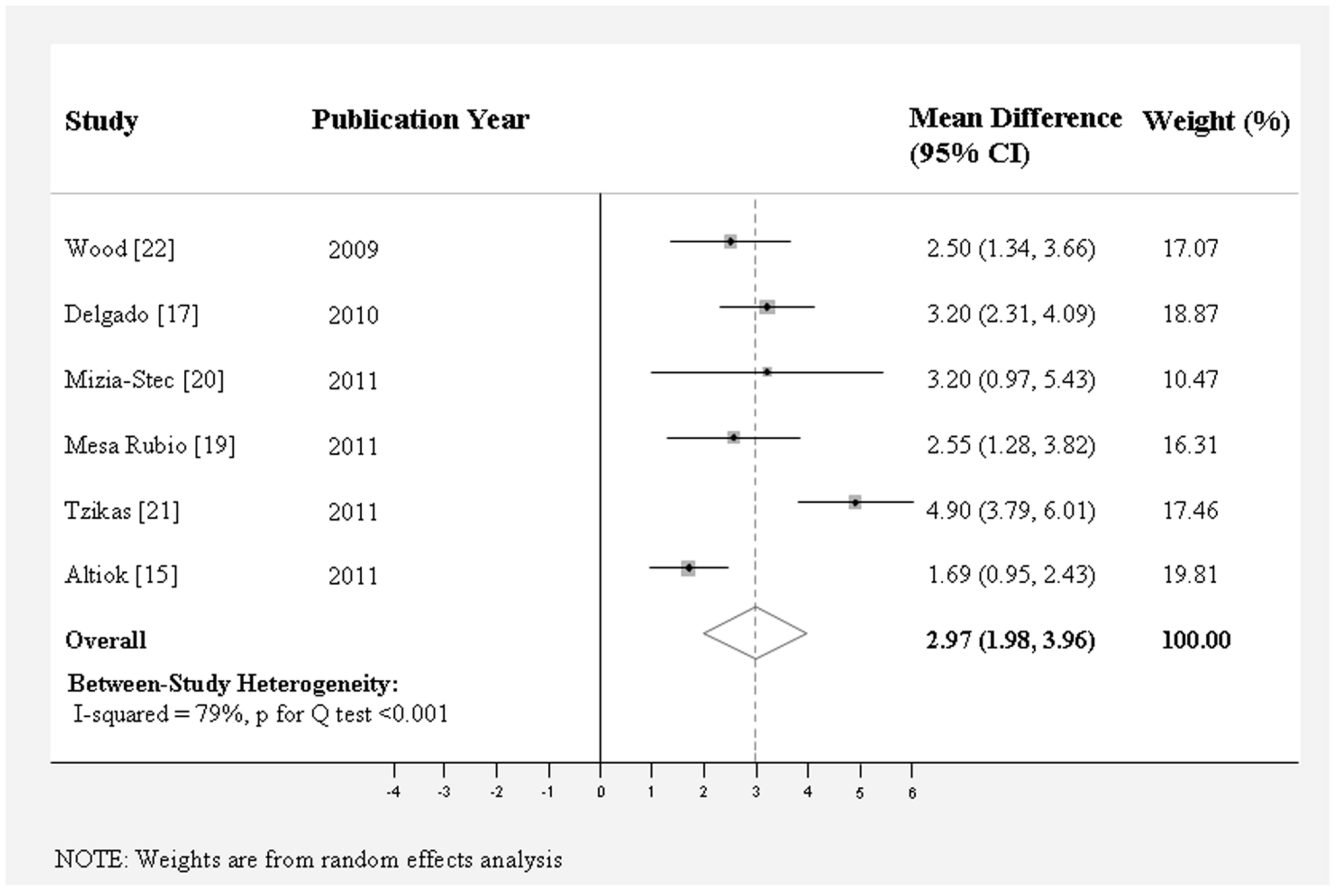
Meta-analysis of the weighted mean difference of aortic annulus diameter measurement between coronal view on MDCT and TTE.

**Figure 3:**
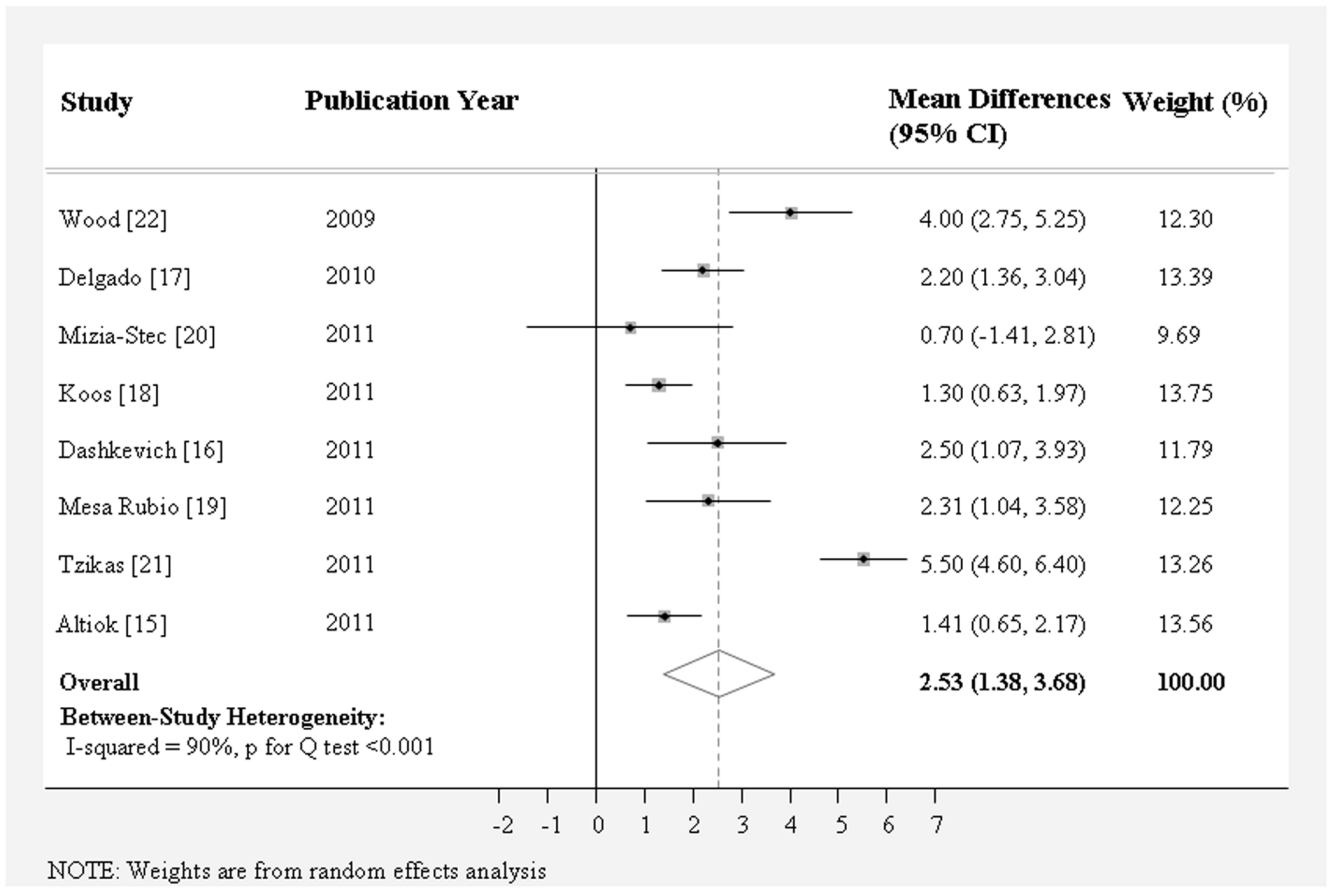
Meta-analysis of the weighted mean difference of aortic annulus diameter measurement between coronal view and sagittal view on MDCT.

**Figure 4:**
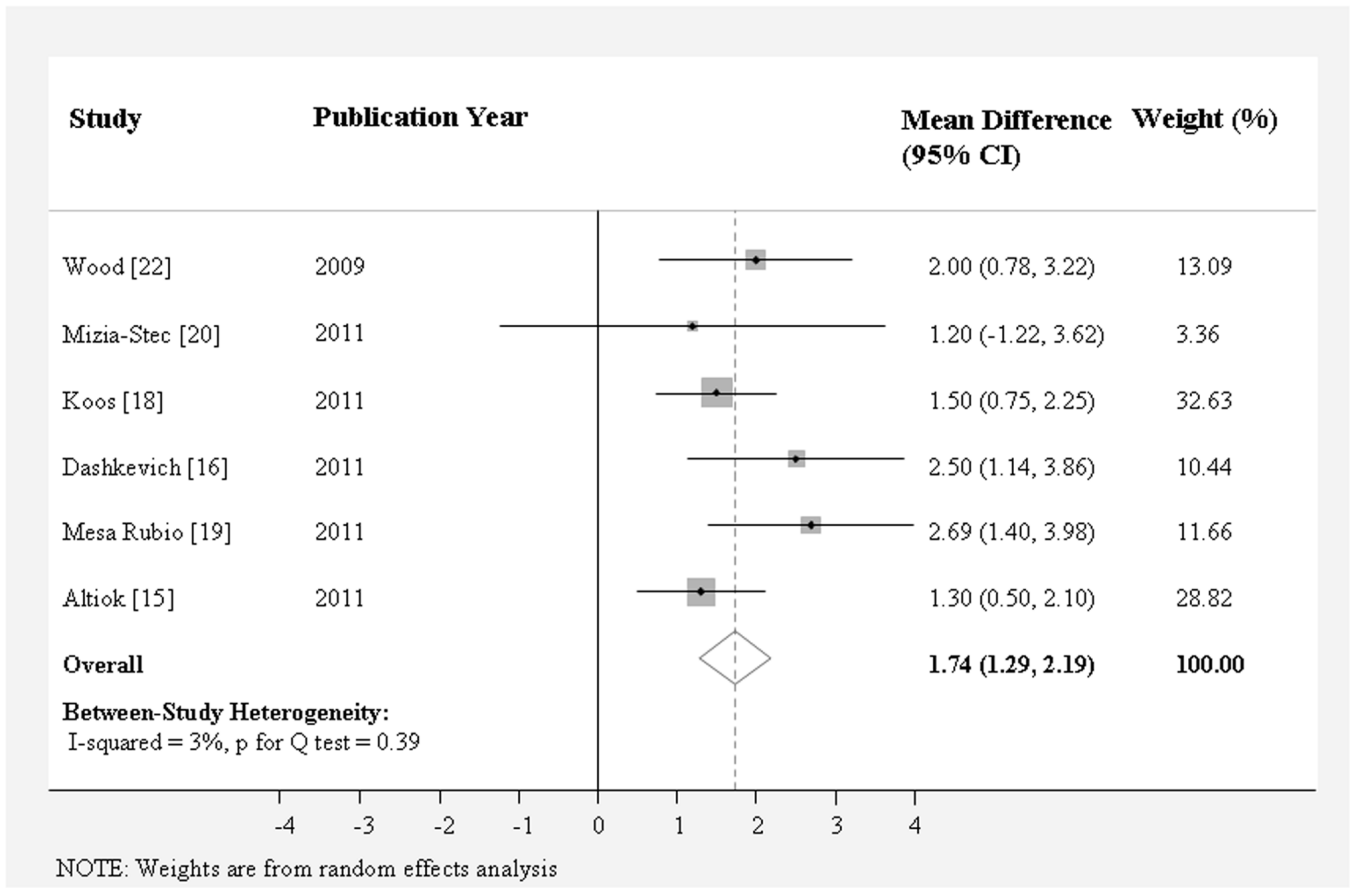
Meta-analysis of the weighted mean difference of aortic annulus diameter measurement between coronal view on MDCT and TEE.

**Figure 5:**
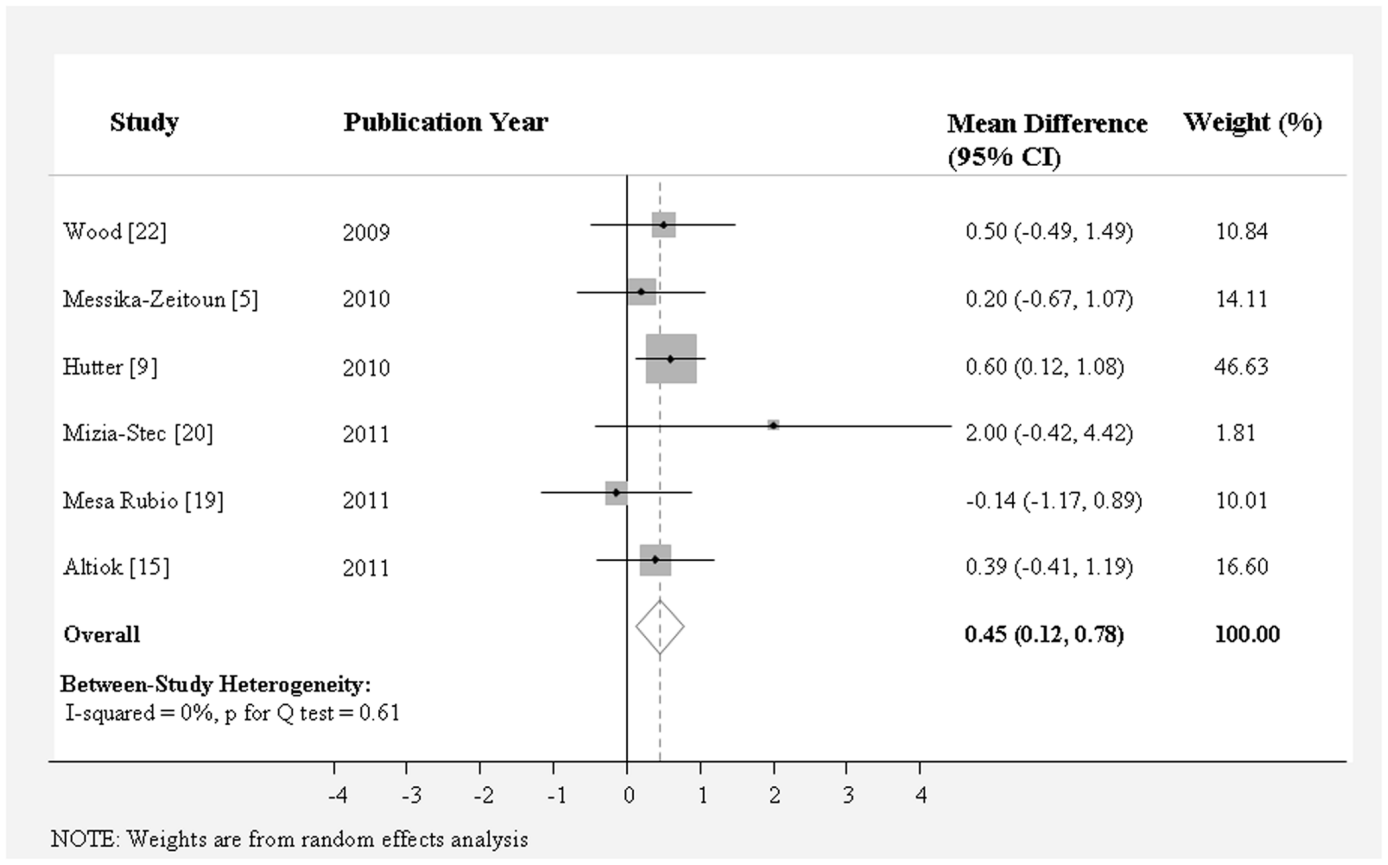
Meta-analysis of the weighted mean difference of aortic annulus diameter measurement between TEE and TTE.

As shown in **Table 2**, aortic annulus diameter measured on coronal view by MDCT (25.3±0.52 mm) was significantly larger than that measured on sagittal view by MDCT (22.7±0.37 mm), TTE (22.6±0.28 mm), or TEE (23.1±0.32 mm). MDCT measurement on coronal view showed the largest aortic annulus diameter and TTE measurement showed the smallest aortic annulus diameter. The weighted mean difference of aortic annulus diameter between these two methods was 2.97 mm (95% CI, 1.98 to 3.96) (Figure 2), followed by that between coronal view and sagittal view by MDCT (2.53 mm; 95% CI, 1.38 to 3.68 mm) (Figure 3), and that between coronal view by MDCT and TEE (1.74 mm; 95% CI, 1.30 to 2.19 mm) (Figure 4) (*P*<0.0001 for all). The mean difference of aortic annulus measurement between TEE and TTE was slight (0.45 mm, *P* = 0.007) (Figure 5). The mean differences between TTE and sagittal view, TTE and three chamber view, TEE and sagittal view, TEE and three chamber view were not significant (*P*>0.05 for all).

**Table 2:** Meta-analysis of the weighted mean differences of aortic annulus measurement between MDCT, TTE, and TEE.

Methods		n	Weighted Mean Differences		Test of Heterogeneity	
			Estimate (95% CI)(mm)	P value	Q-test(P)	I^2^(95% CI)
MDCT Coronal View	vs. Sagittal view	279	2.53(1.38 to 3.68)	<0.0001	<0.001	90(83 to 94)
	vs. TTE	188	2.97(1.98 to 3.96)	<0.0001	<0.001	79(54 to 90)
	vs. TEE	222	1.74(1.30 to 2.19)	<0.0001	0.39	3(0 to 76)
	vs. Surgical	53	2.96(0.51 to 5.40)	0.018	0.04	NA
MDCT Sagittal View	vs. TTE	188	0.17(−0.68 to 1.02)	0.70	0.001	76(45 to 89)
	vs. TEE	222	−0.22(−0.93 to 0.50)	0.55	0.02	62(7 to 84)
	vs. Surgical	53	1.40(−2.80 to 5.60)	0.51	<0.001	NA
						
MDCT Three Chamber View	vs. TTE	232	0.23(−0.19 to 0.64)	0.28	0.47	NA
	vs. TEE	232	−0.30(−0.70 to 0.09)	0.12	0.99	NA
						
TEE	vs. TTE	363	0.45(0.12 to 0.78)	0.007	0.61	0(0 to 75)
	vs. Surgical	53	1.09(−2.63 to 4.81)	0.56	0.002	NA

MDCT: Multi-detector computed tomography, TTE: Transthoracic echocardiography, TEE: Transesophegeal echocardiography; NA, not available for ≤2 studies included in the meta-analysis.

Significant between study heterogeneity were observed for the weighted mean difference of aortic annulus diameter measurement between TTE and coronal view on MDCT (*I* ^2^ = 79%; P for Q test *<*0.001) and that between coronal view and sagittal view on MDCT (*I* ^2^ = 90%; P for Q test <0.001) (Table 2). We conducted a sensitivity analysis to assess the extent to which individual studies with extremely large mean difference on the weighted mean difference. As a result, Tzikas’s study contributed to most of between study heterogeneity [21]. The exclusion of this study appreciably reduced between-study heterogeneity and the final meta-analysis results; the weighted mean differences changed from 2.97 mm (1.98 to 3.96) (*I^2^* = 79% and *P* for Q test <0.001) to 2.50 mm (1.83 to 3.16) (*I^2^* = 44% and *P* for Q test = 0.13) for MDCT coronal view vs. TTE and from 2.53 mm (1.38 to 3.68) (*I^2^* = 90% and *P* for Q test <0.001) to 2.06 mm (1.36–2.76) (*I^2^* = 67% and *P* for Q test = 0.006) for MDCT coronal view vs. sagittal view. Neither did omitting each study from the remaining studies led to almost the same weighted mean differences without substantial changes in between-study heterogeneity.

For aortic annulus diameter measurement between coronal view and sagittal view on MDCT, the Begg’s funnel plot for the visual assessment of publication bias showed that larger mean difference in Tzikas’s study tended to be above the horizontal line, indicating a possibility of publication bias in favor of large difference. For all mean difference measurements, neither the Egger test nor the Begg test showed evidence of publication bias (all p>0.05).

To provide additional information on the accuracy of aortic annulus diameter, we further compared the surgical measurement with those by other imaging modalities, although available data were limited. As a result, the largest mean difference was 2.96 mm between and coronal view on MDCT, followed by the mean difference of 1.40 mm between surgical measurement and sagittal view on MDCT. The smallest mean difference was 1.09 mm between surgical measurement and TEE. Due to small sample sizes, the results should be very suggestive.

## Discussion

Our meta-analysis quantitatively assessed the difference of aortic annulus diameter measurement between MDCT, TTE, and TEE. By synthesizing available data from eligible studies, our results showed that aortic annulus diameter measured on coronal view by MDCT was consistently and significantly larger than that measured on sagittal view by MDCT and that by TTE or TEE. The largest mean difference was seen between coronal view and TTE. Our study provided empirical data for the importance of optimizing the assessment of annular measurement for better selection of valve size for TAVI. Such robust evidence for systematic variability of the aortic annulus measurements between TTE, TEE, and MDCT should be taken into account for choosing prosthetic size.

The largest aortic annulus diameter measured on coronal view by MDCT has been reported by several studies [8], [15], [21]. By achieving more power, our meta-analysis confirmed the result by presenting the mean difference of 2.96 mm between coronal view and TTE. Using two dimensional (2D) TTE and TEE, the aortic annulus diameter is derived from a single diameter measurement on the basis of the assumption of a circular geometry. MDCT could provide three dimensional information of aortic annulus, but it is limited by the radiation exposure and the administration of a contrast agent. From anatomical view, the structure of aortic annulus is not circular but oval. The ellipsoid nature of the aortic annulus is a cause of measurement bias of its diameter assessment and mainly leads to a larger diameter on coronal view than that on sagittal view. The oval shape of the aortic annulus also largely explains the fact that the annulus diameter on the coronal view was consistently larger (from 1.3 mm to 4.0 mm) than that on the sagittal view [8], [22] and that on TEE, because the sagittal view on MDCT is a somewhat similar view with parasternal long-axis view on TTE and long-axis view of the aortic annulus on TEE. The three-dimensional reconstruction of the MDCT images may also contribute to the source of the measured difference. The most important advantage of MDCT is to reconstruct images at any level or plane; however, given the oval shape of the aortic annulus, a minor change of orientation also could lead to significant difference. This process could lead to disparate results between different views on MDCT, and between echocardiography and MDCT. In addition, we could not compare the aortic annulus diameter between the coronal view and three chamber view, due to the absence of relevant data. It seems reasonable to speculate that aortic annulus diameter on coronal view should be larger than that on three chamber view, because three chamber view has the exact same orientation as the parasternal long-axis view on TTE.

Although a 4 mm difference in the annulus diameter measurement between TTE and TEE was shown by one study [6], most studies described the excellent agreement between TTE and TEE [5], [9]. In the present study, we observed no significant differences between TTE and TEE, TTE and sagittal view, TTE and three chamber view, TEE and sagittal view, TEE and three chamber view. The absence of significant difference between those modalities could come from either similar reproducibility of MDCT with TTE and TEE [23], or the small variation of different imaging plane. Both of them can be the potential influencing factor for accurate aortic annulus diameter measurement. In our meta-analysis, between-study heterogeneity in mean differences of aortic annulus diameter was observed but seemed to be influenced by the results from one single study. After excluding this study, the results remained unchanged although between-study heterogeneity disappeared.

Obviously, the differences in aortic annulus measurement between the modalities can cause confusion among operators as to choosing appropriate valve size for TAVI. From clinical perspectives, the method allowing a safe procedure with a low rate of complications should be the best. Koos et al [18] reported the different measurements between TEE and MDCT changed TAVI strategy in a relevant number of patients (22% to 24%). However, it remains uncertain which diameter is more accurate for predicting prosthetic size for TAVI. The results on the agreement of single measurement with final prosthetic size have been mixed [16], [17], [19], [24]. For example, recent studies indicated that TTE measurements might result in undersizing of prosthesis, which is an important determinant for paravalvular aortic regurgitation after TAVI [25], [26]. With the controversial results, Gurvitch R et al [23] used the mean difference between the MDCT measurements and TEE measurements to calculate an “adjusted” sizing model, which reduced the alteration of TAVI by 10% to 12% when unadjusted aortic annulus diameter by MDCT altered the TAVI strategy by 42% to 44%. They also showed that in the cases with residual discrepancies between MDCT and TEE that exceeded these criteria, the frequency of moderate paravalvular aortic regurgitation following TAVI was very high, although future prospective studies are needed to assess the importance and usefulness of the “adjusted” criteria.

In our study, aortic annulus diameter on TEE had the smallest difference with the intra-operative aortic annulus diameter measured by surgical standard method, and coronal view on MDCT showed the biggest mean difference with surgical measurement. However, surgical measurement is made in arrested heart and after excision of the native aortic valve. In percutaneous procedures, the replacement valve is implanted over the native annulus, which is highly calcified in AS patients. Therefore, we could not completely rely on surgical result to evaluate the accuracy of different methods for choosing prosthetic size in TAVI. Although aortic annulus diameter measured by surgical method could not be used as a gold standard, it still is a reflection of the true size of aortic annulus to some degrees.

This study had some limitations. First, aortic annulus diameter measurement on three chamber view by MDCT only was available in 2 studies. At this point, the study power is relatively inadequate to assess the differences between this view and other imaging methods. Second, we could not evaluate the accuracy of TTE, TEE, and MDCT, because the data of the prosthesis size based on good clinical outcomes after TAVI were often absent. Third, measurements of aortic annulus diameter were not made in the same cardiac phase. However, it seems feasible because previous studies showed no significant differences in the diameter of aortic annulus between diastole and systole [27]. Finally, publication bias is evitable in any meta-analysis. Nevertheless, the consistency of the results in almost all included studies indicates that the lack of unpublished data may not substantially affect the pooled results.

In conclusion, aortic annulus diameter measured on coronal view by MDCT was significantly larger than that measured on sagittal view by MDCT and by TTE, TEE. The apparent discrepancy of aortic annulus diameter measurement exists between TTE, TEE and MDCT, and the differences should be taken in to account for choosing prosthetic valve size in TAVI.
